# Rapid, sensitive, and low-cost detection of *Escherichia coli* bacteria in contaminated water samples using a phage-based assay

**DOI:** 10.1038/s41598-022-11468-2

**Published:** 2022-05-11

**Authors:** Luis F. Alonzo, Paras Jain, Troy Hinkley, Nick Clute-Reinig, Spencer Garing, Ethan Spencer, Van T. T. Dinh, David Bell, Sam Nugen, Kevin P. Nichols, Anne-Laure M. Le Ny

**Affiliations:** 1 Intellectual Ventures Laboratory, 14360 SE Eastgate Way, Bellevue, WA 98007 USA; 2 Present Address: Global Health Labs, 14360 Eastgate Way, Bellevue, WA 98007 USA; 3 Present Address: Cell Therapy and Cell Engineering Facility, Memorial Sloan Kettering Cancer Center, New York, NY 10065 USA; 4 Present Address: Independent Consultant, Issaquah, WA 98027 USA

**Keywords:** Lab-on-a-chip, Assay systems, Phage biology

## Abstract

Inadequate drinking water quality is among the major causes of preventable mortality, predominantly in young children. Identifying contaminated water sources remains a significant challenge, especially where resources are limited. The current methods for measuring *Escherichia coli* (*E. coli*), the WHO preferred indicator for measuring fecal contamination of water, involve overnight incubation and require specialized training. In 2016, UNICEF released a Target Product Profile (TPP) to incentivize product innovations to detect low levels of viable *E. coli* in water samples in the field in less than 6 h. Driven by this challenge, we developed a phage-based assay to detect and semi-quantify *E. coli*. We formulated a phage cocktail containing a total of 8 phages selected against an extensive bacterial strain library and recombined with the sensitive NanoLuc luciferase reporter. The assay was optimized to be processed in a microfluidic chip designed in-house and was tested against locally sourced sewage samples and on drinking water sources in Nairobi, Kenya. With this assay, combined with the microfluidic chip platform, we propose a complete automated solution to detect and semi-quantify *E. coli* at less than 10 MPN/100 mL in 5.5 h by minimally trained personnel.

## Introduction

The importance of sustainable access to safe drinking water continues to be critical to reducing poverty and improving the health and well-being of the world’s population. Although progress has been made toward the Sustainable Development Goals and their focus on increasing access to safe water (SDG 6)^1^, the quality of many water supplies remains uncertain. Diarrheal diseases, primarily due to contaminated food and water, continue to be the world’s second leading cause of death in children under five years old, causing 8% of all global deaths, with 80% of the fatalities occurring in South Asia and sub-Saharan Africa^2^. Inspecting water quality for the presence of diarrhea-causing pathogens is vital to the well-being of millions of people.

One of the most significant microbial risks is associated with the ingestion of water contaminated with feces from humans or animals^3^. Though most coliforms are harmless to humans, they are utilized in testing to indicate the potential presence of dangerous pathogens. *E. coli*, in particular, has many ideal features of an indicator organism as it is derived almost exclusively from human and animal feces and includes a few pathogenic strains (e.g., O157:H7)^4^. While the use of fecal indicators (coliforms, thermotolerant coliforms, *E. coli*) has been critiqued since their presence only implies that the risk of pathogen presence has increased, monitoring for the hundreds of known waterborne pathogens remains impractical and is extremely challenging to do rapidly and at low cost^4^. Additionally, information on the quantity of the fecal indicator is essential as the risk of acquiring a waterborne infection increases with the level of contamination.

Acceptable bacterial limits have been defined by WHO^3^, among others, and states that all water intended for drinking must not have any detectable *E. coli* or thermotolerant coliform bacteria in any 100 mL sample, with *E. coli* now being the preferred indicator. The most used methods for detecting coliforms in water samples are membrane filtration, multiple tube fermentation using the Most Probable Number (MPN) method, and presence/absence tests such as the hydrogen sulfide test^5^. While these methods can achieve a sensitivity of 1 CFU/100 mL between $0.5–7.5/test, they are often laborious, require specialized staff training, are difficult to use in the field, and require long incubation times, typically overnight^6^. No test can rapidly detect low levels of viable *E. coli* in water in less than 8 h^7^.

The product innovation team at UNICEF launched the rapid *E. coli* detection project to promote the development of new products to empower communities and government partners with essential information on water quality, allowing them to treat unclean water and identify areas for improvement in water contamination. Accordingly, UNICEF released a Target Product Profile (TPP) to guide industries to develop products that accurately determine fecal contamination as quickly as possible^8^.

A new generation of assays uses engineered bacteriophages to detect pathogens^9, 10^. Phages are viruses that infect and multiply within bacteria and are among the most common and diverse entities in the biosphere. They exhibit specific host ranges due to complex interactions between the phage attachment proteins and the bacterial cell surface^11^. Phages can be genetically modified to carry the genetic information of a reporter protein that is expressed inside the bacteria during phage replication. The use of reporter phages as detection methods has been demonstrated previously to detect pathogens, including *Listeria*^12^*, Mycobacterium*^13^, *Salmonella*^14^, and *E. coli*^15^. Commonly used reporters include, among others, alkaline phosphatase^16^, green fluorescent protein (GFP)^13^, bacterial luciferase (lux systems)^12, 17^, and NanoLuc® luciferase^18–20^. NanoLuc luciferase produces a luminescence signal 100 times stronger than other luciferases and is a smaller protein (19 kDa) that relies on the substrate furimazine to produce a stable, glow-type luminescence^21^.

Here, we use a cocktail of NanoLuc luciferase reporter phages to specifically detect *E. coli* contamination in water. We have developed a very sensitive and rapid assay that allows for the detection of less than 10 MPN *E. coli* in 100 mL of water in 5.5 h, which satisfies the UNICEF TPP requirements and thus enables same-day testing. The phage cocktail has a broad *E. coli* host range that allows using the assay on any water source. Finally, the assay is performed in a microfluidic chip designed to integrate with an automated instrument prototype (a filtration unit, a liquid handling unit, and a detection unit)^22^ for use in the field. The overall cost of the disposable microfluidic device is less than $1, and the associated instrumentation meets the cost targets set forth by the UNICEF TPP^8^, making this platform a suitable alternative to current *E. coli* field test kits.

## Results

### Phage construction

**Figure 1:**
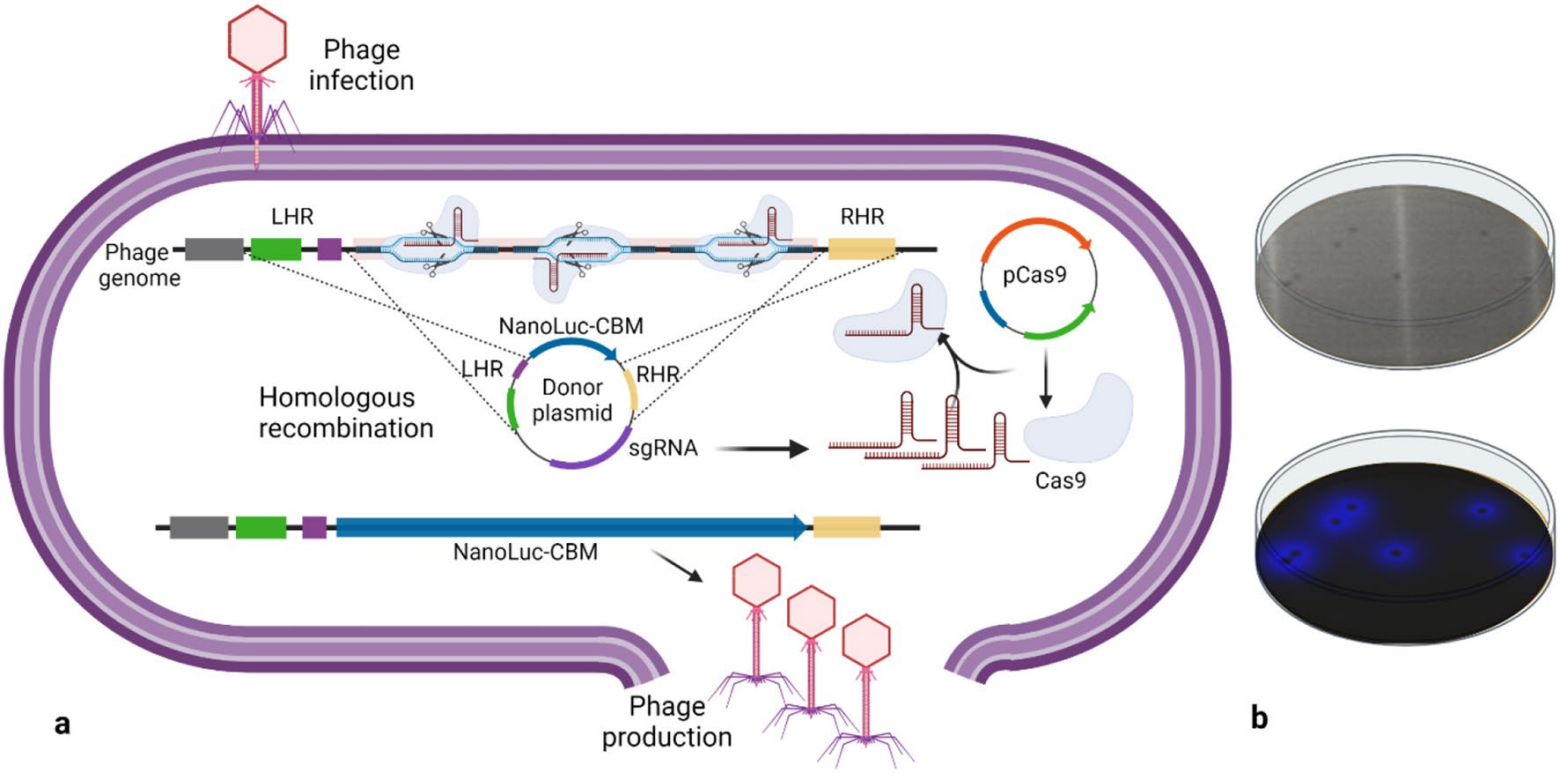
(**a**) Schematic of in-vivo phage recombination using the CRISPR-Cas9 system. Host bacteria cells are transformed with a donor plasmid containing the reporter NanoLuc-CBM flanked with left and right homology regions to the phage (LHR and RHR) as well as selected crRNA and spacer sequences. (**b**) Phage plaque assay shows the presence of plaques in the bacterial lawn (top photo) and NanoLuc activity in the plaques when imaged in a dark room (bottom photo). Created with BioRender.com.

In bacteriophage T4, Hoc (Highly antigenic outer capsid protein) is a highly expressed, non-essential capsid decoration protein^23^, which gene locus has successfully been used to insert proteins into the genome of the phage^24^. Similar proteins have been identified in numerous bacteriophages and double-stranded viruses^25–28^. Genome sequence analysis of our phage library indicated the presence of Hoc homologs in several genomes, including BW-1, HER252, Phi3, RB69, T7, TH07, and TH09, and was used as an insertion site for a NanoLuc reporter fused to a cellulose-binding motif (CBM) expression cassette. As depicted in Fig. 1a, we used CRISPR/Cas9 system^29–31^ for engineering all phages, except T7 and TH09, for which the simpler homologous recombination alone was sufficient to introduce recombinant DNA into the phage genome.

The pCas9 plasmid expresses *S. pyogenes* Cas9 and tracrRNA from its native promoter, and a synthetic sequence downstream of the Lac promoter constitutively expresses the crRNA leader sequence and three spacers flanked by direct repeats. The use of multiple spacers directed towards the hoc locus of a specific phage is two-fold; (1) multiple gRNA directed toward one locus maximize the frequency of Cas9 mediated cleavage of the targeted phage (2) sequence conservation within the hoc locus allows the use of one synthetic sequence to engineer multiple phages (with one or two conserved spacers).

The performance of gRNAs was determined by measuring the efficiency of plaquing (EOP), defined as the log reduction of the phage titer on the strain expressing both Cas9 and gRNA compared to the strain expressing Cas9 only. An efficiency of plaquing of 3 or higher indicated that the CRISPR/Cas9 system is working as desired, and the gRNAs were selected to generate recombinant phages (Table S2). The successful homologous recombination was confirmed by NanoLuc activity in the plaques formed by the recombinant phages and sequencing (Fig. 1b). Surprisingly, one of the NanoLuc expressing phage isolated during the construction of TH09 recombinant phage showed limited homology to the parent TH09 phage sequence. We hypothesize that its original parent phage was present at a very low concentration, underwent recombination in the region homologous to TH09 donor plasmid, and was selected based on NanoLuc activity. The sequence comparison indicated that it is a T4-like phage. This phage was named PJ133.

### Phage purification

Due to the nature of the promoter driving the expression of the reporter, NanoLuc is constitutively expressed during the amplification of the phages to produce high titer reagents and contributes to the background signal in subsequent assays. Hence, to improve the sensitivity of our downstream assays, we developed methods to purify out the reporter.

Phage T7 was purified by taking advantage of the cellulose-binding module (CBM) fused to NanoLuc and using consecutive washes with cellulose-based beads to bind and remove the reporter. This method, which was initially intended to be used for all the recombinant phages, was not effective for other phages. We hypothesized that some of the phages bind to the beads resulting in a low final titer of the purified phage solution and that, in some cases, the reporter binds the beads with low efficiency resulting in a high background signal, as was observed for phage Phi6. Tangential flow filtration (TFF) using cartridges with 500 kDa cutoff membranes proved to be a reliable method to remove the reporter from these phage solutions. Filtration was carried out for 12–18 h until luminescence values plateaued at, or below, 1000 RLU. Filtration efficiency was consistently improved by the addition of detergent (0.05% tween 80) to the media during bacterial cell growth and phage propagation^32^. Concentrations of up to 0.3% Tween 80 did not affect bacterial growth and phage infection (Fig. S1).

### Phage cocktail selection process

**Figure 2:**
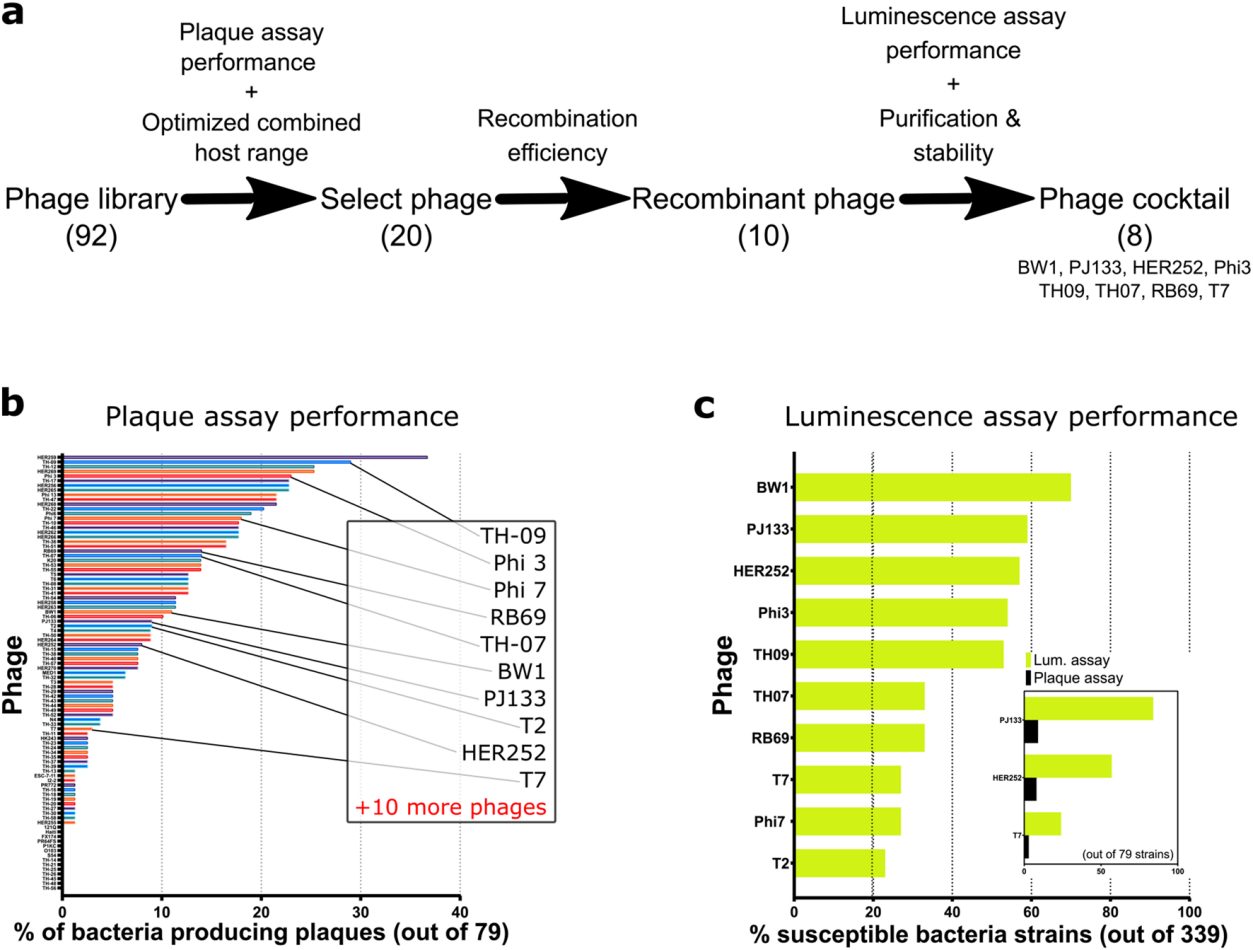
(**a**) Description of the phage selection process: 1. Plaque assay is performed with the entire phage library (92 strains) against a subset of E. coli library (79 strains). Supplemental file 2 contains the detailed plaque plaquing data, 2. Phages are downselected (20 strains) based on the calculated additive host range, 3. Phages are further downselected (10 strains) based on recombination efficiency with NanoLuc reporter, 4. Luminescence assay performance against the entire *E. coli* library (339 strains), purification success, and general stability to determine the final phage cocktail composition (8 strains). (b) % of bacteria producing plaques upon phage infection. (c) % of *E. coli* strains susceptible to recombinant phages determined by luminescence assay. Host range is generally broader on the luminescence assay when compared with the plaque assay results on matched strains (inset shows examples; Fig. S2 has the complete list of phages and Supplemental file 2 shows head-to-head comparison on matching strains).

A phage down-selection process following several steps as described in the schematic in Fig. 2a was used to determine the final composition of the phage cocktail.

For the first step, the host ranges of 92 phages from our library were tested by plaque assay on a subset of *E. coli* strains consisting of the first half of the ECOR library (strains #1 through #36)^33^ and 43 environmental strains. In this experiment, the formation of plaques observed within the lawn of the host strain demonstrated phage replication and propagation within the host and, thus, the susceptibility of the bacterial strain tested. The phages show great variability in host sensitivity, with some exhibiting extensive host ranges. For example, over 25% of host strains demonstrated susceptibility to both TH09 and HER259 phages, while 14 phages did not plaque on any of the 79 bacteria strains tested (Fig. 2b and Tables S4 and S5 and Supplementary file 2). Based on the breadth of phage individual host coverages and the calculated additive coverage of phages used in combination, 20 phages were selected to be genetically engineered.

The NanoLuc enzyme reporter was successfully introduced into the genomes of twelve phages, and their host ranges were assessed using the luminescence assay on the entire *E. coli* strain in-house library (339 strains described in methods and listed in Tables S2 and S3). For the luminescence assay, which is more conducive to high-throughput screening, a signal from wells containing both an *E. coli* host and a recombinant phage was compared to control wells containing the recombinant phage only (Fig. 2c). Positive host susceptibility was recorded in any wells with luminescence above mean background values +3 Standard Deviations (SD). When comparing the results obtained for plaque and luminescence assays on matching strains, we noticed that the host range based on the luminescence assay is generally broader and shows additional host-range sensibility (Supplementary file 2). For example, this observation is evident for phages HER252, BW-1, and PJ133, for which the host range increased by 49%, 57% and 75%, respectively (Fig. 2c, inset and Fig. S2 shows the rest of the phages). A comparison of the host range measured by plaque assay between engineered and wild-type phage was tested for phage T2 and did not show host range differences suggesting that the change in host range is not due to manipulating the phage’s genome. Additionally, the host range of engineered phage PJ133 is broader when assessed by luminescence than by plaque assay (Supplementary file 2).

The list of selected phages was further refined based on several factors such as lysis time, reporter production efficiency, and stability of the purified phage solution, with the goal to combine phages with high titers (> 10^9^ PFU/mL) and low luminescence background that induce the expression of a high luminescence signal relatively fast (< 3 h) and that are stable at 4 °C. For example, reporter phage Phi7 and T7 have similar luminescence-based host ranges; however, T7 yielded a positive luminescence signal two hours faster and was selected over phage Phi7. The titer of phage T2 decreased by a couple of logs over the course of a few weeks during storage at 4 °C and was not pursued as a candidate for the cocktail. Similarly, the titer of phage T6 was found to decrease during centrifugation steps and was also deprioritized in favor of other phages with better stability during purification. Finally, phage Phi6 was removed early in the process due to poor purification efficiency resulting in high luminescence background levels.

At the end of the phage engineering and selection process, a phage cocktail was developed, containing eight phages: BW-1, HER252, Phi3, PJ133, RB69, T7, TH07, and TH09. The host range testing result based on the luminescence assay on our entire *E. coli* strain library for the selected phages is shown in Fig. 2c. Host range coverage varies from 27% (T7) to 70% (BW-1) and adds up to 90% of the library if we assume no negative interferences from the different phages.

### Optimization of the luminescence assay

Laboratory strain ATCC 25922 was choosen to determine the duration of phage infection to achieve the lowest bacterial limit of detection, using the cocktail of eight phages as it is a listed QC strain by US EPA for analyzing drinking water. The strain is sensitive to phages HER252, BW1, and PJ133, with phage BW1 inducing the stronger luminescence signal (Fig. S3). When the phages are in cocktail, we have observed the signal to be dominated by the phage inducing the strongest signal. A serial dilution of bacterial cells in stationary phase was first incubated in a 96-well plate in growth media for 2 h at 37 °C to allow for recovery and ready the cells for protein expression before the addition of the phage cocktail.

**Figure 3:**
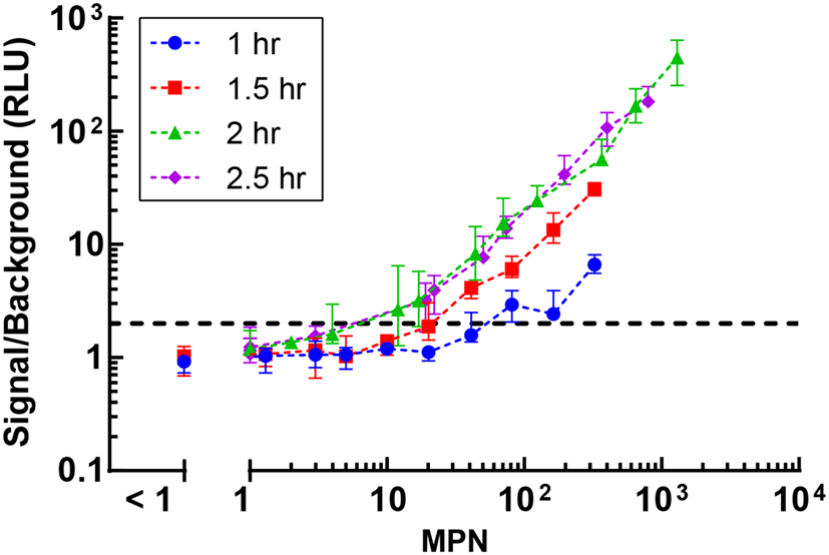
Median luminescence signal of 8 replicates with interquartile range normalized to phage only background as a function of MPN/well for 1, 1.5, 2, and 2.5-h phage incubations using ATCC 25922 and the 8-phage cocktail. MPN values were determined by Quanti-Tray/2000, in triplicate. The dashed line represents values at 2× background.

For the phage infection step, the bacteria were incubated with the phages for 1, 1.5, 2 or 2.5 h before concentrating the expressed NanoLuc reporter and measuring the resulting luminescence signal. The signal is normalized to the background luminescence generated by the phage cocktail only and compared for the different infection times at several cell concentrations to determine the corresponding limit of detection for each condition (Fig. 3). The MPN (Most Probable Number) value represents the number of cells that were added per well as determined by a Quanti-Tray/2000 system, in triplicate. As contact time between phage and bacteria cells increases, the limit of signal detection above background decreases. For example, a 1-, 1.5-, and 2-h incubation period results in a signal twice over background from 60, 20, and 8 cells, respectively. Additional incubation time after 4 h total incubation does not result in an increase in signal over the range of cell numbers tested.

Note that, with strain ATCC 25922, there does not seem to be an advantage nor a disadvantage in incubating the bacteria with the phages for more than 2 h as we observe the signal plateauing after 2 h. Thus, for subsequent experiments with field samples that contain uncharacterized bacterial populations and within the microfluidic device^22^, we will use a phage incubation time of 3 h to ensure that all bacteria have time to produce the enzymatic reporter.

### Field evaluation of preliminary phage cocktail

**Figure 4:**
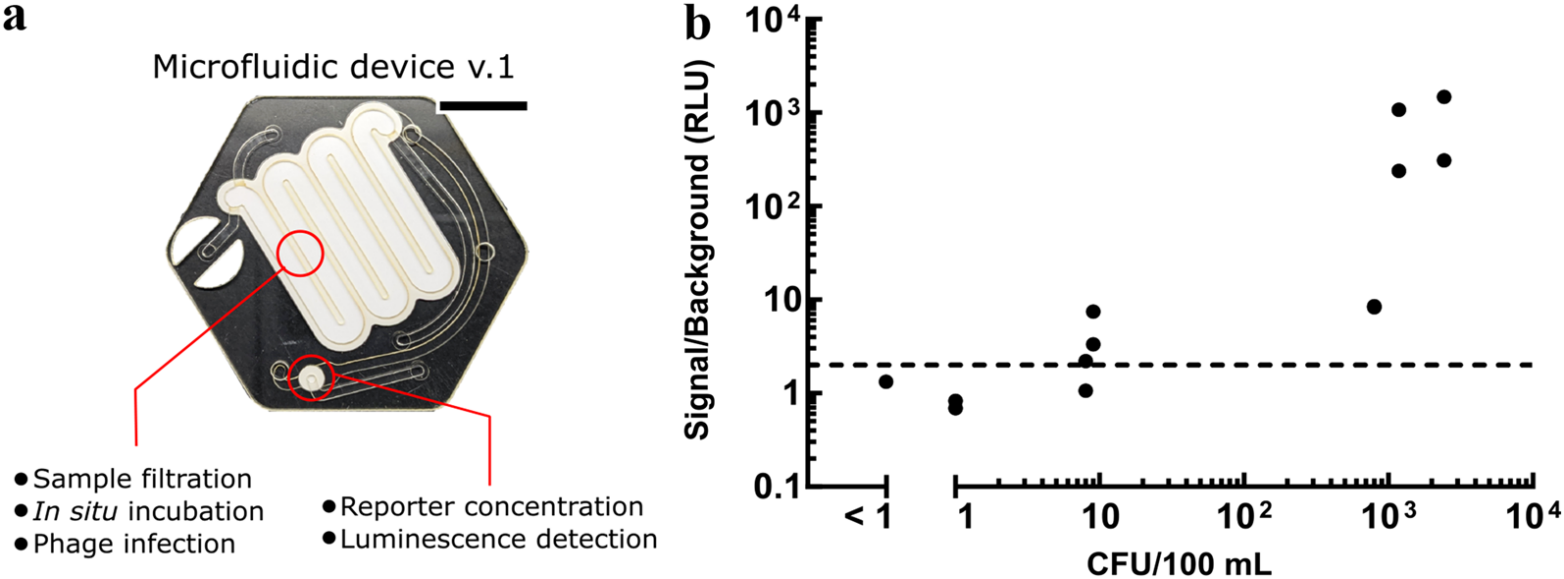
Validation of preliminary phage cocktail assay on local sewage samples. (**a**) First prototype of the microfluidic device for the detection of E. coli in water samples. Scale bar: 10 mm (**b**) Luminescence signal normalized to phage only background of water samples obtained using a cocktail of 4 phages (Phi3, Phi7, RB69, and T7) and processed through the microfluidic chip device. Water samples were collected from the Ngong river flowing through the city of Nairobi. CFU values were determined using a membrane filtration field kit. The dashed line represents values at 2× background.

We assembled an *E. coli* library with a wide geographical (Tables S2 and S3) and genetic diversity to select the phages for the cocktail^33^. Yet, we wanted to assess early in the cocktail formation whether this phage selection process was biased towards bacterial populations specific to our collection. Thus, at the time of this field evaluation, our cocktail was at an early stage of development and contained four phages, namely Phi3, Phi7, RB69, and T7. The performance of the preliminary phage cocktail was tested in combination with an early microfluidic chip prototype designed to replace the manual steps of the assay, as described in our previous work^22^ (Fig. 4a). The chip integrates a first membrane where bacteria are captured, infected with phage, and the reporter is expressed. A second membrane in the device is where the expressed reporter is concentrated and detected. The performance of the phage-based microfluidic assay was tested on local field samples collected from the Ngong River flowing through the city of Nairobi, Kenya. The samples were, in general, very turbid and highly contaminated. Dilutions of the samples were prepared in sterile distilled water before processing to assess a range of bacterial concentrations (Fig. 4b).

Fluid additions to the microfluidic chip were performed manually using disposable silicone rubber fixtures surrounding the inlet/outlet ports providing an interface for pipette tips containing needed reagents and tubbing connected to the vacuum source. A vacuum source connected to outlet ports was used to control the fluidic flow within the microfluidic device. In this experiment, the processed volume of water was 10 mL, which we have shown in a previous study to yield equivalent results to the same number of bacteria in a 100 mL sample volume^22^. The assay was run for a total of 5 h, divided into a 2-h bacteria recovery period followed by a 3-h phage infection to ensure enough time for the cells to produce the NanoLuc reporter enzyme.

From our results, we observe a clear signal over background for both samples tested containing 9 CFU and for one out of two samples containing 8 CFU (Fig. 4). These results demonstrate that with a 4-phage cocktail, we can already measure less than 10 *E. coli* CFU per 100 mL of water. Also, note that the filtration test kit used as the reference method here detects the presence of thermotolerant coliforms and not *E. coli* specifically, representing only about 80% of thermotolerant coliforms^34, 35^. Additionally, there is about 15 to 20 times more non-*E. coli* bacteria in the samples, as determined by total plate count performed by AgriQ Quest Laboratory in Nairobi, where we processed the samples, and the relative abundance of the genus Escherichia/Shigella in bacterial population of fecal sludge is estimated to be 0.1–0.8%^36^. This indicates that the assay does not detect non-*E.coli* bacteria and that the host range of the phages is specific to *E. coli*. Similar results were observed for experiments run on the river samples in a 96-well plate format, which allowed us to test a larger number of samples and replicates (Fig. S4). Based on these preliminary results, which confirmed our *E. coli* library was sufficiently diverse, we further pursued the development of the phage cocktail to design a potentially more robust assay with an increased host range and lower limit of detection. Note that at the time of testing, phage Phi7 was part of the cocktail but later found to not increase the performance of the overall cocktail and was removed in favor of better performing phages.

### Performance of final phage cocktail assay on local sewage samples

**Figure 5:**
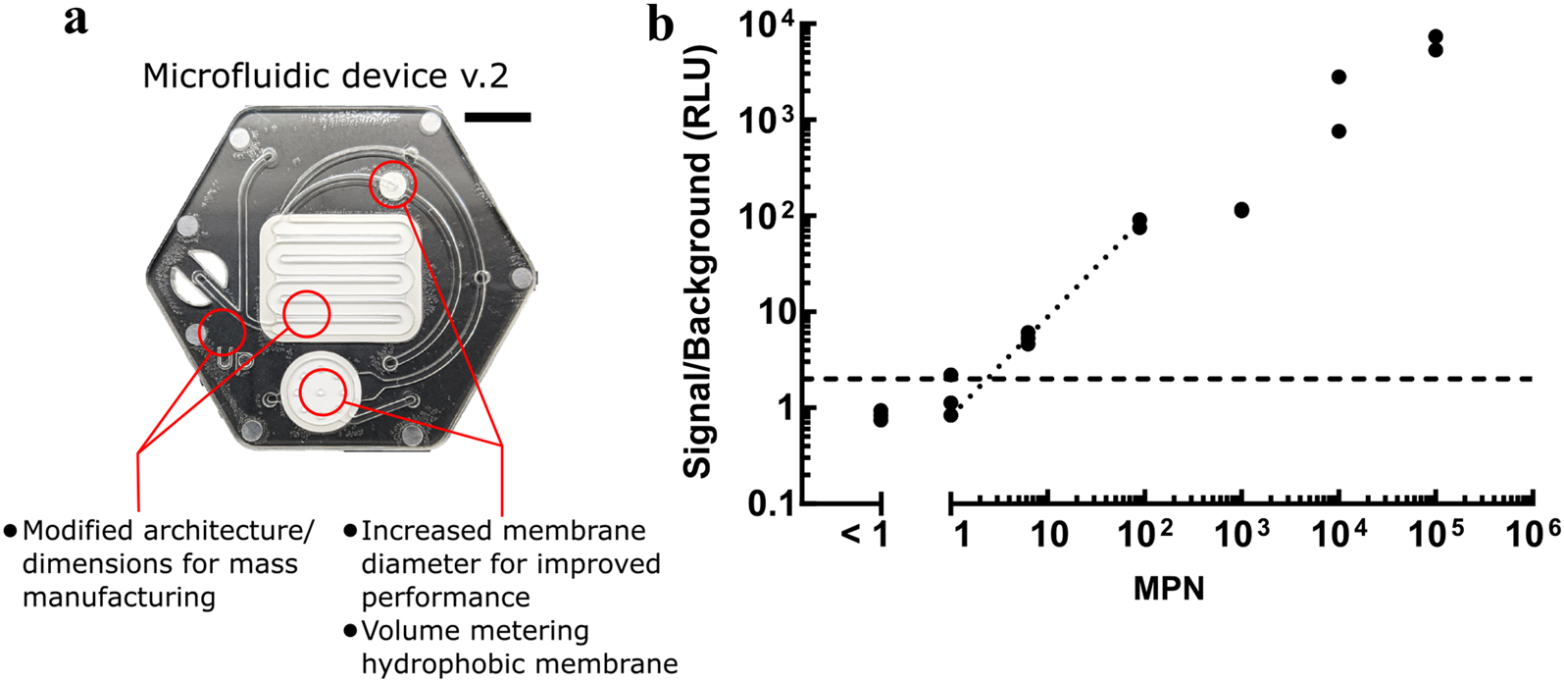
Validation of final phage cocktail assay on local sewage samples. (**a**) Second prototype of the microfluidic device for the detection of E. coli in water samples. Scale bar: 10 mm (**b**) Luminescence signal normalized to phage only background of sewage samples obtained using the 8-phage cocktail and processed through the microfluidic chip device. MPN values were determined with a Quanti-Tray/2000 assay. The dotted line shows a linear regression performed on a subset of the data, between MPN values of 1 and 100. The dashed line represents values at 2× background.

The completed phage cocktail, containing 8 phages, was challenged with sewage samples collected at a local water treatment plant (King County South Water Treatment plant in Renton, WA USA). As for our field evaluation of the partial cocktail, the assay was carried out using an in-house designed microfluidic chip, this time with an updated version shown in Fig. 5a and further described in previous work^22^. The new version of the microfluidic device contains all the same functional units of the previous version; however, small modifications were made to allow for an injection-molded manufacturing process, which considerably reduced the overall cost of the test. Several dilutions of the sewage sample were prepared either in duplicate (MPN values > 1000) or in triplicate (MPN values < 1000) to generate a range of cell concentrations for testing (Fig. 5b).

The data presented here aggregates the results obtained with sewage samples collected on two different days and for which no difference was observed. The x-value < 1 corresponds to a negative Quanti-Tray/2000 assay that has an assigned Most Probable Number (MPN) value of less than 1/100 mL as per the manufacturer’s directions. For these samples, the luminescence signal measured is comparable to the background signal from phage-only controls. At an MPN value of 1 (95% Confidence interval (CI) = 0–3.7 listed by the manufacturer), the phage assay detects a signal more than 2 times over the background in only 1 out of 3 samples. At very low cell concentrations, the bacterial population follows a Poisson distribution^37^, which explains that some of the diluted samples may contain cells while others may not. At an MPN value of 6 however, with a 95% CI = 2.3–12.1 indicating that most samples contain bacteria, a signal around 5 times above background is detected in all three replicates. We performed a linear regression in the lower cell number range only (< 100) to demonstrate a simple calibration curve for our assay. This allows us to determine bacteria number ranges based on the luminescence response measured with the assay: < 1, between 1 and 10, between 10 and 100, and > 100.

## Discussion

Rapid, low-cost, and fieldable methods to indicate the presence of pathogens in water sources in low-resource settings are needed to identify contaminated sources. The methods currently available to governmental and non-profit organizations that often perform testing to quantify *E. coli* contamination typically involve long incubation periods and complicated processes. Additionally, they require specialized training, or samples must be transported and processed in a laboratory. Unfortunately, this is often not feasible due to the lack of nearby, necessary infrastructures.

The design of a rapid and low-cost test to detect low levels of viable *E. coli* bacteria in a large volume of water (100 mL) has proven challenging to the scientific community. Previous efforts have had success in the development of reporter phages for this application^18, 38, 39^. Unfortunately, the methods use lab strains and phages with very limited host ranges and assay protocols, making them unsuitable for field applications. To address this gap, we screened a library of 92 phages, including over 50 novel phages isolated for this work, and validated the performance of a microfluidic device that we previously developed in our lab on real-world environmental water samples from various locations. Our overall method combines:

- A phage cocktail with an extensive host range specific to *E. coli.*
- An enzymatic reporter, NanoLuciferase, which, combined with NanoGlo substrate, produces a very bright luminescent signal allowing for a low limit of detection.
- A disposable microfluidic device combining a bacteria concentration step, in-situ phage infection, and concentration of expressed reporter to increase the sensitivity of the assay before detection.

As a first step towards assay development, we identified and engineered several phages that were selected based on various characteristics (amenability to be recombined with the NanoLuc reporter, stability during and post purification processes, final phage purity and titer, time to generate a signal, and intensity of luminescence signal resulting from infection) to be combined in a phage cocktail. The assembly of an *E. coli* strain library to select phages against was based on genetic and geographic diversity with a bias for strains isolated in limited-resource settings. The phage selection process was based on empirical data as we did not find ways to predict phage stability or the success of purification processes. During host range determination, we observed that, for all the phages tested, the host range determined by luminescence assay is broader than when determined by plaque assay. We hypothesize this difference to be a result of abortive infections^40^, in which an infected bacterial cell dies prematurely and thereby prevents the production and further propagation of mature phages. While a sustained infection is not generated and plaques are not observed, the reporter enzyme is produced at levels high enough to be detected. Alternatively, modifications to the plaque assay (e.g., lower concentration of soft agar overlay) could impact plaque morphology and will be further explored to investigate if agreement can be reached between plaque and luminescence assay results.

The expression of the reporter enzyme is controlled by a constitutive promoter and is thus produced during phage propagation. Consequently, we devoted significant development efforts in purification methods to produce phage reagents with high titers and low reporter concentration that do not generate substantial background signal. Only phage T7 could be efficiently purified using successive rounds of cellulose beads purification. With this method, the cellulose-based beads bind to the cellulose-binding motif (CBM) fused to the NanoLuc reporter which can then be removed from the solution. All other phages would suffer titer drops, or residual luminescence would plateau at unacceptably high values due to a significant portion of NanoLuc proteins not binding to the beads and remaining enzymatically active. Size exclusion-based methods such as tangential flow filtration proved to be effective in these cases and allowed us to process large volumes.

In assays measuring viable bacteria, there is usually a trade-off between time to detection and limit of detection as bacteria going through a period of recovery and outgrowth generate a higher signal. Bacteria cells collected from environmental water samples are expected to be in a dormant state as the water environment is low on nutrients and at a lower temperature than the digestive tract of mammals. When stationary phase bacteria cells are placed in a nutrient-rich medium at an optimal temperature, the cells first enter a lag phase, which is an initial time period during which the cells adjust to their new environment and are preparing for exponential growth^41^. Thus, for cells to effectively express the reporter enzyme, we want to ensure that the cells have entered the exponential growth phase when proteins are expressed at high levels. Based on previous studies performed within the microfluidic device^22^, a 1 to 2-h period of cell metabolic recovery was the minimum amount of time necessary to ensure effective phage infection. Since we expect the water samples to contain various bacterial strains with potentially different recovery times, we chose a 2-h incubation period before phage infection. Then, we determined the time to observe maximum signal generation after phage infection. Using the cocktail of 8 phages and a lab strain, we observe maximum signal generation after a 2-h phage infection that plateaus at longer infection times. Since we do not observe a negative effect from longer infection times and anticipate that the phages infect an uncharacterized bacterial population from environmental samples, we chose an infection time period of 3 h, resulting in a total assay incubation time of 5 h.

We evaluated the performance of the assay and validated our phage selection method early in the development by testing the assay on real-world samples in Nairobi, Kenya, a location where this product would be intended to be used. This evaluation is critical to properly assessing the cocktail performance and ensuring the validity of our phage selection method. At the time of this evaluation, the phage cocktail contained four phages (Phi3, Phi7, RB69, and T7). With just 4 phages, the assay performed very well on these environmental samples, detecting less than 10 *E. coli* MPN/100 mL. Additionally, we observed little signal over background for samples containing 1 or less thermotolerant coliform CFU, indicating that the assay is specific for *E. coli*. Together, the results demonstrated that our in-house *E. coli* library allows us to select phages with host ranges that are not only specific to our *E. coli* library and that will infect a wide range of bacteria. Based on these results, we pursued the development of the phage cocktail and the assay. Note that no inhibitory effects were observed when incrementally adding new phages to the cocktail; thus, a cocktail with a higher number of phages is desirable as it increases the chances of finding sensitive *E. coli* strains in a water sample.

Water samples were collected from a local water treatment plant to validate further the performance of the phage-based assay and the composition of the phage cocktail on complex bacterial samples and the microfluidic chip device. These results show that the method detects *E. coli* bacteria cells down to 6 *E. coli* MPN, in 5.5 h (total time including incubation periods, filtration, and reporter concentration steps) as measured against the Quanti-Tray/2000 reference method. Additionally, the signal varies linearly with the bacterial count, which can be fitted to a calibration curve. This allows to determine contamination levels, < 1 *E. coli* MPN/100 mL (no to very low contamination), 1–10 *E. coli* MPN/100 mL (low contamination), 10–100 *E. coli* MPN/100 mL (high contamination), and > 100 *E. coli* MPN/100 mL (very high contamination). This calibration curve is dependent on the quality of the phage reagents (titer, purity) and would have to be determined for each phage cocktail production lot.

To be used with the microfluidic chip device, our lab designed an instrument allowing a user with minimal training to run the phage-based assay. The design of the instrument and software were made available in our previous publication by Alonzo et al.^22^. Together with this instrument, we are proposing a complete solution to specifically measure less than 10 viable *E. coli* MPN/100 mL, in the field, in 5.5 h while differentiating relevant *E. coli* bacterial contamination levels below 1/100 mL, between 1 and 10/100 mL, between 10 and 100/100 mL, and above 100/100 mL, at costs similar to existing field-testing kits.

To facilitate remote access, prototypes can fit in a small carry-on luggage-sized Pelican case, are lightweight, and can be battery operated. Further development of the instrumentation prototype to add automation and increase throughput will enable testing in remote locations by minimally trained users. Additional development efforts on reagent stability and packaging, specifically phage and substrate solution lyophilization, are of interest to enhance the prospect of a portable system. Testing of the assay on natural waters in a variety of locations will be necessary to refine phage selection to produce a robust cocktail composition and achieve high sensitivity and specificity. Alternatively, directed genetic modifications of phage tail components involved in the initial binding event have successfully expanded the host range^42^. These strategies can also be utilized to design specific bacteriophages aimed at detecting other pathogens and indicators in other liquid samples such as urine or beverages.

## Methods

### Bacterial strains and phages

Our bacterial collection consists of 339 *E. coli* strains, including 72 strains representing the ECOR library^33^ purchased from Michigan State University, 116 strains purchased from the *E. coli* reference center at Penn State University College of Agricultural Sciences, and 151 strains isolated from water samples collected in low- and middle-income countries (LMIC) from various water sources (rivers, wells, and stagnant waters). Collected water samples from LMIC were filtered through 0.45 µm membranes (Millipore HAWP04700) that were then placed on MLGA selective media (Sigma # 39734) plates and incubated at 30 °C for 4 h followed by 18 h at 37 °C. Up to 10 *E. coli* colonies per water source were collected and streaked to isolate them from possible contaminants. Selected strains were grown in TSB (trypic soy broth) and stored at − 80 °C.

Our phage collection consists of 92 lytic phages, including 39 phages purchased from Université Laval, QC and 53 coliphages (labeled TH-#) isolated from wastewater samples collected at a local water treatment plant (King County, Department of Natural Resources and Parks, Wastewater treatment division) as described previously^43^. Briefly, raw sewage (25 mL) was mixed with 2 × LB (Luria broth) (25 mL) and an overnight culture of *E. coli* host cells (500 µL) in a 150 mL flask. After overnight incubation (37 °C, 250 rpm), centrifugation (3000 × *g*, 10 min), and filtration (0.22 µm) of the culture, the supernatant yielded a phage lysate that was subsequently plaque purified three times on the original host strain to produce a homogeneous phage stock. The genomes of isolated phages were fully sequenced in an Illumina Miseq as described elsewhere^43^. The phages were deposited in the Nugen collection at Cornell University.

### Host range by plaque assay

The host range of each phage from our collection was determined by double overlay plaque assay (LB-agar and 0.8% soft LB agar overlay)^44^ on a limited set of bacteria consisting of 36 strains from the ECOR library (ECOR #1 to #36) and 43 bacteria isolated from environmental water samples.

### Phage engineering, propagation, and purification

Restriction enzymes, T4 DNA ligase, NEBuilder HiFi DNA Assembly Master Mix, and NEB 5-alpha competent *E. coli* cells were ordered from New England Biolabs, Inc. Oligonucleotides used in this study were synthesized by IDT. The plasmid expressing *S. pyogenes* Cas9 (pCas9) was kindly gifted by Sam Nugen, Cornell University. The plasmid expressing *E. coli* codon-optimized NanoLuc® Luciferase gene linked to a C-terminal CBM2a^45^ via a flexible linker (pUC57-NanoLuc) and the plasmids expressing 3 × gRNA, targeting specific regions in the phage, were synthesized by GenScript. Phage sequences were either downloaded from NCBI or sequenced in-house on an Illumina MiSeq as described elsewhere^43^. The sequences were assembled using Geneious software (version 11.1.5, Biomatters Ltd., Auckland, New Zealand)^46^ and were compared to other genomes using NCBI BLAST^47^, PSI-BLAST^48^, and Clustal Omega^49^.

Phage engineering was performed in vivo, using the CRISPR-Cas9 system as described previously in a homologous recombination proficient strain^50^. Phage-specific donor plasmid was generated by two-step cloning in NEB 5-alpha. First, 600–1500 bp of Left and Right Homology Regions (LHR and RHR) were amplified using phage DNA as template and were cloned upstream and downstream of NanoLuc-CBM cassette using NEBuilder HiFi assembly mix protocol. Functional crRNA leader sequence and 3 × gRNA cassette were then cloned into their respective phage-specific plasmid to construct the final donor plasmid. All primer pairs and gRNA sequences used are listed in Tables S1 and S2. 100 ng pCas9 and phage-specific donor plasmid were sequentially electroporated in DY331 using standard conditions. Successfully transformed DY331 strain were transiently induced for pro-recombination lambda red genes as described elsewhere^51^. The strains were then infected with the appropriate phage (titers from 10^4^ to 10^9^) for in vivo recombination and poured on soft agar.

Nano-Glo® (Promega, Madison, WI), prepared according to manufacturer’s instructions, was uniformly sprayed on the plates and imaged to screen for glowing recombinant phages plaques. Recombinant phages were picked in PBS buffer (1 mL), and tenfold serial dilutions were plated on their respective host three times to purify the recombinant phages. All the recombinant phages were confirmed by sequencing. For T7, the reporter phage was constructed by assembly of overlapping PCR fragments of T7 phage genome and reporter expression cassette. Each adjacent fragment has homology ≥ 30 bp. Primers used to amplify T7 genome, and insertion site of reporter cassette is as described previously^52^. Recombinant TH07 phage was selected by plating the wild-type phage on strain harboring allelic exchange substrate and screening for glowing plaques as described above.

Recombinant phages were propagated in liquid culture according to previous methods^53^. Overnight cultures of host cells (NEB 5-alpha) were diluted (1:100) in TSB media supplemented with Tween 80 (0.05%). Host cells were incubated (37 °C, 250 rpm, OD_600_ = 0.15), inoculated with a phage stock solution (MOI = 0.1), and further incubated (37 °C, 250 rpm, 3–5 h) to produce a high titer phage lysate. The lysate was centrifuged (3750 × *g*, 10 min) to pellet cellular debris, and the supernatant was filtered (0.22 µm, EMD Millipore, Burlington, MA, USA) before storage at 4 °C. The phage solutions were purified to remove NanoLuc expressed during propagation. Cellulose beads (Avicel, PH-101, ~ 50 µm, Millipore Sigma, Darmstadt, Germany) were added (5 g per 100 mL, 30 min, 250 rpm, 37 °C) to phage lysates to bind the reporter and removed via filtration (0.22 µm) before tangential flow filtration (TFF). Partially purified phage lysates (50 mL) were added to SM buffer (50 mM Tris–HCl, 8 mM magnesium sulfate, 100 mM sodium chloride, 0.01% gelatin, pH 7.5) with 0.05% Tween 80 (450 mL) and placed on the Minimate TFF system (Pall Biotech, New York, NY, USA) with a 500 kDa cutoff membrane. Continuous diafiltration was performed until luminescence values of the retentate plateaued. The purified phage solution was then concentrated to its original volume, recovered from the membrane, and stored at 4 °C. The purification procedure for phage T7 consisted of consecutive cellulose washes (5 g per 100 mL, sterile filtration, repeat 5–7 times) until luminescence values plateaued. Phage stock solutions at high titer (> 10^9^ PFU/mL) were treated with sodium azide (0.05%) to create a storage phage stock solution. Finally, a phage cocktail solution was prepared by mixing stock solutions of eight phages in TSB (each at final concentration 1:80 from stock) and stored at 4 °C until ready to use. The phage titers were stable for at least 6 months.

### Host range by Luminescence assay

The host range of the recombinant phages was evaluated on the full *E. coli* library (339 strains) in a multi-well plate assay. Overnight *E. coli* culture (7.5 µL) was added to TSB (117.5 µL) in a standard 96-well plate and incubated (37 °C, 1.5 h, 100 rpm). A phage solution in TSB buffer (25 µL) at high titer (> 10^8^ PFU/mL) was added to appropriate wells (final well composition: TSB with 10 mM MgCl_2_, 2 mM CaCl_2_, 50 mM Tris–HCl pH 7.5). The plate was incubated for an additional 3 h, after which 100 µL of the culture was transferred to a white 96-well plate, mixed 1:1 with NanoGlo substrate, and incubated at room temperature for 3 min before reading using a plate reader (BioTek Synergy H1, 1 mm read height, 1 s integration).

### Optimization of phage infection time

An overnight culture of ATCC 25922 was serially diluted in PBS to yield solutions containing between 1 and 1000 *E. coli* bacteria/mL, as measured by the Most Probable Number (MPN) technique using a Quanti-Tray/2000 system, in triplicate. Each dilution (40 µL) was added in eight replicate wells of a 96-well PVDF filter plate (Corning) and filtered to collect the bacteria. TSB (90 µL) was then added to each well, and the plate was incubated (37 °C, 2 h). The phage cocktail solution (10 µL) was added to appropriate wells, and the plate was incubated for 1, 1.5, 2, or 2.5 h. After incubation, the solution was filtered through, and the NanoLuc reporter was collected. The filtrate solution was added to a 384-well cellulose filter white plate (Pall, Acroprep) and again filtered to collect and concentrate the reporter on the nitrocellulose membrane. Nano-Glo substrate (10 µL) was added to each well, and luminescence was measured as described previously.

### Assay performance using microfluidic device

A bacteria detection microfluidic device was designed in-house and outsourced for manufacturing (HC 1101-001, Hochuen Medical Technology, Shenzhen, China). A complete description of the microfluidic device can be found in a separate study^22^. Briefly, the device consists of two interconnected functional features: (1) a low protein-binding filtration area designed to isolate and grow bacteria from environmental water samples followed by in situ cocktail phage infection and (2) a cellulose-based collection and activation area for phage-induced luminescence reporter enzyme.

### *Escherichia coli* detection in river and sewage samples

Water samples (≥ 100 mL) in Nairobi, Kenya were collected from Ngong River along the Mukuru Kayaba area and transported to a nearby laboratory. Sewage samples (≥ 100 mL) were collected from a local facility. Turbidity was measured using the HTTURB® turbidity meter (Wagtech Projects Ltd., Thatcham, UK), and samples were diluted to 1 NTU for processing with the microfluidic device. Samples were further serially diluted in distilled water to generate a range of cell concentrations for testing. The desired dilution (100 mL) was added to TSB media (10 mL) on a syringe connected to the microfluidic chip using a custom-built filtration assembly. During the filtration process, vacuum was applied to the outlet port causing any particles larger than 0.45 µm—including *E. coli* cells—to be captured on a PVDF membrane. To increase the metabolic activity of the isolated cells, the serpentine channel chamber above the PVDF membrane was filled with TSB media (250 µL) containing Tween 20 (0.1%). The chip was then incubated in a laboratory incubator (37 °C, 2 h). Media was then replaced with phage cocktail solution at > 10^7^ (250 µL). The chip was further incubated (37 °C, 3 h) to allow for phage infection. Vacuum was then applied to transfer the produced NanoLuc-CBM enzyme from the PVDF membrane to the nitrocellulose (NT) capture membrane. Finally, Nano-Glo substrate (75 µL) was added to the chamber below the NT membrane. Luminescence was read immediately, using a custom-built detection assembly with a photomultiplier tube (ET Enterprises, Ltd., Uxbridge, UK)^22^. The Aquasafe® WSL50 Pro membrane filtration kit (Wagtech Projects Ltd., Thatcham, UK) was used to quantify CFU’s within the river samples, and the Quanti-Tray/2000 system (IDEXX, Westbrook, ME) was used to determine MPN in sewage samples.

## Supplementary Information

Supplementary Information 1.

Supplementary Information 2.
