# Supplementary material for: Rapid, sensitive, and low-cost detection of Escherichia coli bacteria in contaminated water samples using a phage-based assay
Source: Sci Rep. 2022 May 11;12:7741. doi: 10.1038/s41598-022-11468-2 (PMC9095594; doi:10.1038/s41598-022-11468-2)
Supplement: Supplementary file 1 — Supplementary Information 1. [file 41598_2022_11468_MOESM1_ESM.docx]

# Supplementary Information:

| Phages | Primer sequence | | |
| --- | --- | --- | --- |
| BW-1 | LHR | For | GGGATAACAGGGTAAACATGTTTGCCCGCCAAAAATTGGCCACCAATTCCGCCAGGTTTC |
|  |  | Rev | CTATAGTGAGTCGTATTAATTTGAATTCCTGTAAAAGTCATAAGTTATCCTTA |
|  | RHR | For | CTTCCAAAGATTGGTTGGTGGGTTATGGATGAAATCTTATGTTCATCCTCTTCCACATCG |
|  |  | Rev | AGCGGGCAGTGAAAGGAAGGCCCATTTATAACCAACTTTTGGACAACCGCTGAAGTTTCC |
| HER252 | LHR | For | AGGGATAACAGGGTAAACATGTTTGCCCGCCAAAAATTGGCATCCCAGTGGCCATATTTG |
|  |  | Rev | TATAGTGAGTCGTATTAATTTGGCGGCCGCCAATTCTTGGAACTCCTGTTGGGGTTTTAGGAGTTATATC |
|  | RHR | For | GCTTCCAAAGATTGGGGTTGGTGGGTTATGGATGAAATCCAAAAAATGACTGAAGAAGG |
|  |  | Rev | AGCGGGCAGTGAAAGGAAGGCCCATTTATAACCAACTTTTGGACAACCGCTGAAGTTTCC |
| Phi3 | LHR | For | AGGGATAACAGGGTAAACATGTTTGCCCGCCAAAAATTGGGCCGCAGATGATGTGGATGG |
|  |  | Rev | TATAGTGAGTCGTATTAATTTGGCGGCCGCCAATTCTTGGGGAACACCAAAGACCGTAGT |
|  | RHR | For | GCTTCCAAAGATTGGGGTTGGTGGGTTATGGATGAAAGCAACTTTAACAGCATCTGTTAC |
|  |  | Rev | TAATTAATCGATTCTAGACTAGTTTATAACCAACTTTTGGCGAAGCCATTGACCGACTTG |
| RB69 | LHR | For | AGGGATAACAGGGTAAACATGTTTGCCCGCCAAAAATTGGACCATCCCATTGCCCATAGC |
|  |  | Rev | AGTGAGTCGTATTAATTTGGCGGCCGCCAATTCTTGGATGTTTGTGAACCTCCAATAACC |
|  | RHR | For | CTTCCAAAGATTGGTTGGTGGGTTATGGATGAAATCACTACCTGCATCGTTACACTCTTC |
|  |  | Rev | CGGGCAGTGAAAGGAAGGCCCATTTATAACCAACTTTTGGCACCGTAGTCAACACCTTTC |
| TH07 | LHR | For | AGGGATAACAGGGTAAACATGTTTGCCCGCCAAAAATTGGACCATCCCATTGCCCATAGC |
|  |  | Rev | TATAGTGAGTCGTATTAATTTGGCGGCCGCCAATTCTTGGGGAACACCAAAGACCGTAGT |
|  | RHR | For | GCTTCCAAAGATTGGGGTTGGTGGGTTATGGATGAAAGCAACTTTAACAGCATCTGTTAC |
|  |  | Rev | TAATTAATCGATTCTAGACTAGTTTATAACCAACTTTTGGCGAAGCCATTGACCGACTTG |
| TH09 | LHR | For | TAGGGATAACAGGGTAAACATGTTTGCCCGACTAAGCCGAATG |
|  |  | Rev | CTATAGTGAGTCGTATTAATTTGAATTCCTGTAAAAGTCATAAGTTATCCTTA |
|  | RHR | For | CTTCCAAAGATTGGTTGGTGGGTTATGGATGAAATCTTATGTTCATCCTCTTCCACATCG |
|  |  | Rev | TGGAAAGCGGGCAGTGAAAGGAAGGCCCATGCTAGCGACAGAATCTAAATCATTTGGC |

Table S1. Primers used for the construction of donor plasmids.

| Phages | gRNA sequences | Efficiency of plaquing (Titer on strain expressing Cas9 and gRNA/Titer on Wild Type strain) |
| --- | --- | --- |
| BW-1 | TCCACATCGTACTTCAGCTTATATCTGGTG | 3.5 x10^-5^ |
|  | GTGCCATTGATACGTAGCAGATGCACCTGG |  |
|  | TGAAACCAAGCAGTTTACTGCTGCACCCAG |  |
| HER252 | TACAGTTGATATAACTCCTAAAACACCGAC | 5.1x10^-4^ |
|  | CAGCTTACATCTGGTGCGGTTGGTGGGTTA |  |
|  | CAACCTGATGGAGCATCTGCTACGTATCAG |  |
| Phi3 | TGCACTAGAGACATTTGCAGTTAAAGTAAT | 7.3x10^-5^ |
|  | AGTTCCACCAACAGTAGTATTAACTGATGG |  |
|  | CTTCTGCTACGTATAAATTCACTCCAACCG |  |
| RB69 | CACTGCTACACCTGCAGGTGAAACTCCAGA | 4.9x10^-5^ |
|  | ACCGCACCAGATGTAAGCTGAAGTACGATG |  |
|  | CTTCTGCTACGTATAAATTCACTCCAACCG |  |
| TH07 | CACTGCTACACCTGCAGGTGAAACTCCAGA | 8.9x10^-3^ |
|  | ACCGCACCAGATGTAAGCTGAAGTACGATG |  |
|  | CTTCTGCTACGTATAAATTCACTCCAACCG |  |

Table S2. gRNAs used for the construction of donor plasmids.

| Penn State Accession Number | IVL ID | Country of Origin | Species/Sample Source |
| --- | --- | --- | --- |
| 92.0142 | PS-01 | India | Human/Feces |
| 92.0143 | PS-02 | India | Human/Feces |
| 92.0144 | PS-03 | India | Human/Feces |
| 92.0145 | PS-04 | India | Human/Feces |
| 92.0146 | PS-05 | India | Human/Feces |
| 92.0147 | PS-06 | India | Human/Feces |
| 92.0148 | PS-07 | India | Human/Feces |
| 92.0149 | PS-08 | India | Human/Feces |
| 92.015 | PS-09 | India | Human/Feces |
| 92.0151 | PS-10 | India | Human/Feces |
| 92.0152 | PS-11 | India | Human/Feces |
| 92.0153 | PS-12 | India | Human/Feces |
| 92.0154 | PS-13 | India | Human/Feces |
| 92.0155 | PS-14 | India | Human/Feces |
| 92.0156 | PS-15 | India | Human/Feces |
| 92.0157 | PS-16 | India | Human/Feces |
| 92.0158 | PS-17 | India | Human/Feces |
| 92.0159 | PS-18 | India | Human/Feces |
| 92.016 | PS-19 | India | Human/Feces |
| 92.0161 | PS-20 | India | Human/Feces |
| 92.0162 | PS-21 | India | Human/Feces |
| 92.0163 | PS-22 | India | Human/Feces |
| 92.0164 | PS-23 | India | Human/Feces |
| 92.0165 | PS-24 | India | Human/Feces |
| 92.0166 | PS-25 | India | Human/Feces |
| 93.0458 | PS-26 | Kenya | Human/Blood |
| 93.0459 | PS-27 | Kenya | Human/Blood |
| 93.046 | PS-28 | Kenya | Human/Blood |
| 93.0461 | PS-29 | Kenya | Human/Blood |
| 93.0464 | PS-30 | Kenya | Human/Blood |
| 93.0465 | PS-31 | Kenya | Human/Blood |
| 93.0466 | PS-32 | Kenya | Human/Blood |
| 93.0468 | PS-33 | Kenya | Human/Blood |
| 93.047 | PS-34 | Kenya | Human/Blood |
| 93.0471 | PS-35 | Kenya | Human/Blood |
| 93.0472 | PS-36 | Kenya | Human/Blood |
| 93.0474 | PS-37 | Kenya | Human/Blood |
| 93.0475 | PS-38 | Kenya | Human/Blood |
| 93.0477 | PS-39 | Kenya | Human/Blood |
| 93.0478 | PS-40 | Kenya | Human/Blood |
| 99.0484 | PS-41 | India | Human |
| 99.0485 | PS-42 | India | Human |
| 99.0486 | PS-43 | India | Human |
| 99.0487 | PS-44 | India | Human |
| 99.0488 | PS-45 | India | Human |
| 99.0489 | PS-46 | India | Human |
| 99.049 | PS-47 | India | Human |
| 99.0491 | PS-48 | India | Human |
| 99.0492 | PS-49 | India | Human |
| 99.0493 | PS-50 | India | Human |
| 99.0494 | PS-51 | India | Human |
| 99.0495 | PS-52 | India | Human |
| 99.0496 | PS-53 | India | Human |
| 99.0497 | PS-54 | India | Human |
| 99.0498 | PS-55 | India | Human |
| 99.0499 | PS-56 | India | Human |
| 99.05 | PS-57 | India | Human |
| 99.0501 | PS-58 | India | Human |
| 99.0502 | PS-59 | India | Human |
| 99.0503 | PS-60 | India | Human |
| 99.0504 | PS-61 | India | Human |
| 99.0505 | PS-62 | India | Human |
| 99.0506 | PS-63 | India | Human |
| 99.0507 | PS-64 | India | Human |
| 94.0012 | PS-65 | Zambia | Human/Intestine |
| 94.0013 | PS-66 | Zambia | Human/Intestine |
| 94.0014 | PS-67 | Zambia | Human/Intestine |
| 94.0016 | PS-68 | Zambia | Human/Intestine |
| 94.0017 | PS-69 | Zambia | Human/Intestine |
| 94.0020 | PS-70 | Zambia | Human/Intestine |
| 94.0022 | PS-71 | Zambia | Human/Intestine |
| 94.0025 | PS-72 | Zambia | Human/Intestine |
| 94.0028 | PS-73 | Zambia | Human/Intestine |
| 94.0029 | PS-74 | Zambia | Human/Intestine |
| 94.0031 | PS-75 | Zambia | Human/Intestine |
| 94.0036 | PS-76 | Zambia | Human/Intestine |
| 94.0040 | PS-77 | Zambia | Human/Intestine |
| 94.0041 | PS-78 | Zambia | Human/Intestine |
| 94.0043 | PS-79 | Zambia | Human/Intestine |
| 94.0059 | PS-80 | Zambia | Human/Intestine |
| 94.0061 | PS-81 | Zambia | Human/Intestine |
| 94.0071 | PS-82 | Zambia | Human/Intestine |
| 94.0073 | PS-83 | Zambia | Human/Intestine |
| 94.0075 | PS-84 | Zambia | Human/Intestine |
| 91.0975 | PS-85 | Saudi Arabia | Human/Feces |
| 91.0976 | PS-86 | Saudi Arabia | Human/Feces |
| 91.1007 | PS-87 | Saudi Arabia | Human/Feces |
| 91.1008 | PS-88 | Saudi Arabia | Human/Feces |
| 91.1009 | PS-89 | Saudi Arabia | Human/Feces |
| 91.1017 | PS-90 | Saudi Arabia | Human/Feces |
| 91.1025 | PS-91 | Saudi Arabia | Human/Feces |
| 91.1026 | PS-92 | Saudi Arabia | Human/Feces |
| 91.1048 | PS-93 | Saudi Arabia | Human/Feces |
| 10.0174 | PS-94 | Philippines | Chicken |
| 10.0175 | PS-95 | Philippines | Chicken |
| 10.0176 | PS-96 | Philippines | Chicken |
| 10.0177 | PS-97 | Philippines | Chicken |
| 10.0178 | PS-98 | Philippines | Chicken |
| 10.0179 | PS-99 | Philippines | Chicken |
| 10.0180 | PS-100 | Philippines | Chicken |
| 10.0181 | PS-101 | Philippines | Chicken |
| 10.0182 | PS-102 | Philippines | Chicken |
| 10.0183 | PS-103 | Philippines | Chicken |
| 88.0168 | PS-104 | Mexico | Human/Feces |
| 88.0184 | PS-105 | Mexico | Human/Feces |
| 88.0185 | PS-106 | Mexico | Human/Feces |
| 88.0474 | PS-107 | Mexico | Human/Feces |
| 88.0476 | PS-108 | Mexico | Human/Feces |
| 88.1202 | PS-109 | Mexico | Human/Feces |
| 88.1218 | PS-110 | Mexico | Human/Feces |
| 88.1223 | PS-111 | Mexico | Human/Feces |
| 88.1225 | PS-112 | Mexico | Human/Feces |
| 88.1232 | PS-113 | Mexico | Human/Feces |
| 15.0661 | PS-114 | India | Water |
| 15.0662 | PS-115 | India | Water |
| 15.0663 | PS-116 | India | Water |

Table S3. E. coli isolates selected from the E. coli Reference Center at Penn State College of Agricultural Sciences.

| IVL ID | Country of origin | Sample type |
| --- | --- | --- |
| 01-01 | Uganda | Well |
| 01-02 | Uganda | Well |
| 01-03 | Uganda | Well |
| 01-04 | Uganda | Well |
| 01-05 | Uganda | Well |
| 01-06 | Uganda | Well |
| 01-07 | Uganda | Well |
| 01-08 | Uganda | Well |
| 01-09 | Uganda | Well |
| 01-10 | Uganda | Well |
| 01-11 | Uganda | Well |
| 04-01 | Uganda | Well |
| 04-02 | Uganda | Well |
| 04-03 | Uganda | Well |
| 04-04 | Uganda | Well |
| 04-05 | Uganda | Well |
| 04-06 | Uganda | Well |
| 04-07 | Uganda | Well |
| 04-08 | Uganda | Well |
| 04-09 | Uganda | Well |
| 04-10 | Uganda | Well |
| 06-01 | Thailand | Sewage |
| 06-02 | Thailand | Sewage |
| 06-03 | Thailand | Sewage |
| 06-04 | Thailand | Sewage |
| 06-05 | Thailand | Sewage |
| 06-06 | Thailand | Sewage |
| 06-07 | Thailand | Sewage |
| 06-08 | Thailand | Sewage |
| 06-09 | Thailand | Sewage |
| 06-10 | Thailand | Sewage |
| 06-11 | Thailand | Sewage |
| 06-12 | Thailand | Sewage |
| 06-13 | Thailand | Sewage |
| 06-14 | Thailand | Sewage |
| 06-15 | Thailand | Sewage |
| 06-16 | Thailand | Sewage |
| 06-17 | Thailand | Sewage |
| 06-18 | Thailand | Sewage |
| 06-19 | Thailand | Sewage |
| 06-20 | Thailand | Sewage |
| 06-21 | Thailand | Sewage |
| 06-22 | Thailand | Sewage |
| 06-23 | Thailand | Sewage |
| 06-24 | Thailand | Sewage |
| 06-25 | Thailand | Sewage |
| 07-01 | Thailand | Sewage |
| 07-02 | Thailand | Sewage |
| 07-03 | Thailand | Sewage |
| 07-04 | Thailand | Sewage |
| 07-05 | Thailand | Sewage |
| 07-06 | Thailand | Sewage |
| 07-07 | Thailand | Sewage |
| 07-08 | Thailand | Sewage |
| 07-09 | Thailand | Sewage |
| 07-10 | Thailand | Sewage |
| 07-11 | Thailand | Sewage |
| 07-12 | Thailand | Sewage |
| 07-13 | Thailand | Sewage |
| 07-14 | Thailand | Sewage |
| 07-15 | Thailand | Sewage |
| 07-16 | Thailand | Sewage |
| 09-01 | Ghana | Reservoir |
| 09-02 | Ghana | Reservoir |
| 09-03 | Ghana | Reservoir |
| 09-04 | Ghana | Reservoir |
| 09-05 | Ghana | Reservoir |
| 09-06 | Ghana | Reservoir |
| 09-07 | Ghana | Reservoir |
| 09-08 | Ghana | Reservoir |
| 09-09 | Ghana | Reservoir |
| 09-10 | Ghana | Reservoir |
| 09-11 | Ghana | Reservoir |
| 09-12 | Ghana | Reservoir |
| 09-13 | Ghana | Reservoir |
| 09-14 | Ghana | Reservoir |
| 09-15 | Ghana | Reservoir |
| 09-16 | Ghana | Reservoir |
| 09-17 | Ghana | Reservoir |
| 09-18 | Ghana | Reservoir |
| 10-01 | Kenya | Lake |
| 10-02 | Kenya | Lake |
| 10-03 | Kenya | Lake |
| 10-04 | Kenya | Lake |
| 10-05 | Kenya | Lake |
| 10-06 | Kenya | Lake |
| 10-07 | Kenya | Lake |
| 10-08 | Kenya | Lake |
| 10-09 | Kenya | Lake |
| 10-10 | Kenya | Lake |
| 10-11 | Kenya | Lake |
| 11-01 | Ecuador | Reservoir |
| 11-02 | Ecuador | Reservoir |
| 11-03 | Ecuador | Reservoir |
| 11-04 | Ecuador | Reservoir |
| 11-05 | Ecuador | Reservoir |
| 11-06 | Ecuador | Reservoir |
| 11-07 | Ecuador | Reservoir |
| 11-08 | Ecuador | Reservoir |
| 12-01 | Colombia | River |
| 12-02 | Colombia | River |
| 12-03 | Colombia | River |
| 12-04 | Colombia | River |
| 12-05 | Colombia | River |
| 12-06 | Colombia | River |
| 12-07 | Colombia | River |
| 12-08 | Colombia | River |
| 12-09 | Colombia | River |
| 12-10 | Colombia | River |
| 12-11 | Colombia | River |
| 12-12 | Colombia | River |
| 12-13 | Colombia | River |
| 12-14 | Colombia | River |
| 12-15 | Colombia | River |
| 12-16 | Colombia | River |
| 12-17 | Colombia | River |
| 12-18 | Colombia | River |
| 12-19 | Colombia | River |
| 12-20 | Colombia | River |
| 12-21 | Colombia | River |
| 12-22 | Colombia | River |
| 12-23 | Colombia | River |
| 12-24 | Colombia | River |
| 12-25 | Colombia | River |
| 13-01 | Peru | River |
| 13-02 | Peru | River |
| 13-03 | Peru | River |
| 13-04 | Peru | River |
| 13-05 | Peru | River |
| 13-06 | Peru | River |
| 13-07 | Peru | River |
| 13-08 | Peru | River |
| 13-09 | Peru | River |
| 13-10 | Peru | River |
| 13-11 | Peru | River |
| 13-12 | Peru | River |
| 13-13 | Peru | River |
| 13-14 | Peru | River |
| 14-01 | Salomon Islands | Washing well |
| 15-01 | Salomon Islands | Pond |
| 15-02 | Salomon Islands | Pond |
| 15-03 | Salomon Islands | Pond |
| 15-04 | Salomon Islands | Pond |
| 15-05 | Salomon Islands | Pond |
| 15-06 | Salomon Islands | Pond |
| 15-07 | Salomon Islands | Pond |
| 15-08 | Salomon Islands | Pond |
| 15-09 | Salomon Islands | Pond |
| 15-10 | Salomon Islands | Pond |
| 15-11 | Salomon Islands | Pond |
| 16-01 | Salomon Islands | Drinking well |

Table S4. E. coli isolates collected from environmental water samples.

| *IVL isolated Phage ID* | *Genbank Accession No.* | *% bacteria producing plaques (out of 79)* |
| --- | --- | --- |
| TH-06 | [MT446386](https://www.ncbi.nlm.nih.gov/nuccore/MT446386) | 10 |
| TH-07 |  | 14 |
| TH-08 |  | 13 |
| TH-09 | [MT446387](https://www.ncbi.nlm.nih.gov/nuccore/MT446387) | 29 |
| TH-10 | [MT446388](https://www.ncbi.nlm.nih.gov/nuccore/MT446388) | 18 |
| TH-11 | [MT446389](https://www.ncbi.nlm.nih.gov/nuccore/MT446389) | 3 |
| TH-12 | [MT446390](https://www.ncbi.nlm.nih.gov/nuccore/MT446390) | 25 |
| TH-13 | [MT446391](https://www.ncbi.nlm.nih.gov/nuccore/MT446391) | 1 |
| TH-14 |  | 0 |
| TH-15 | [MT446392](https://www.ncbi.nlm.nih.gov/nuccore/MT446392) | 8 |
| TH-16 | [MT446393](https://www.ncbi.nlm.nih.gov/nuccore/MT446393) | 1 |
| TH-17 |  | 23 |
| TH-18 |  | 1 |
| TH-19 |  | 1 |
| TH-20 | [MT446394](https://www.ncbi.nlm.nih.gov/nuccore/MT446394) | 1 |
| TH-21 | [MT446395](https://www.ncbi.nlm.nih.gov/nuccore/MT446395) | 0 |
| TH-22 | [MT446396](https://www.ncbi.nlm.nih.gov/nuccore/MT446396) | 20 |
| TH-23 | [MT446397](https://www.ncbi.nlm.nih.gov/nuccore/MT446397) | 3 |
| TH-24 | [MT446398](https://www.ncbi.nlm.nih.gov/nuccore/MT446398) | 3 |
| TH-25 | [MT446399](https://www.ncbi.nlm.nih.gov/nuccore/MT446399) | 0 |
| TH-26 | [MT446400](https://www.ncbi.nlm.nih.gov/nuccore/MT446400) | 0 |
| TH-27 | [MT446401](https://www.ncbi.nlm.nih.gov/nuccore/MT446401) | 1 |
| TH-28 | [MT446402](https://www.ncbi.nlm.nih.gov/nuccore/MT446402) | 5 |
| TH-29 | [MT446403](https://www.ncbi.nlm.nih.gov/nuccore/MT446403) | 5 |
| TH-30 | [MT446404](https://www.ncbi.nlm.nih.gov/nuccore/MT446404) | 1 |
| TH-31 |  | 13 |
| TH-32 | [MT446405](https://www.ncbi.nlm.nih.gov/nuccore/MT446405) | 6 |
| TH-33 | [MT446406](https://www.ncbi.nlm.nih.gov/nuccore/MT446406) | 4 |
| TH-34 | [MT446407](https://www.ncbi.nlm.nih.gov/nuccore/MT446407) | 3 |
| TH-35 | [MT446408](https://www.ncbi.nlm.nih.gov/nuccore/MT446408) | 3 |
| TH-36 |  | 16 |
| TH-37 | [MT446409](https://www.ncbi.nlm.nih.gov/nuccore/MT446409) | 3 |
| TH-38 | [MT446410](https://www.ncbi.nlm.nih.gov/nuccore/MT446410) | 8 |
| TH-39 |  | 3 |
| TH-40 | [MT446411](https://www.ncbi.nlm.nih.gov/nuccore/MT446411) | 8 |
| TH-41 | [MT446412](https://www.ncbi.nlm.nih.gov/nuccore/MT446412) | 13 |
| TH-42 | [MT446413](https://www.ncbi.nlm.nih.gov/nuccore/MT446413) | 5 |
| TH-43 | [MT446414](https://www.ncbi.nlm.nih.gov/nuccore/MT446414) | 5 |
| TH-44 | [MT446415](https://www.ncbi.nlm.nih.gov/nuccore/MT446415) | 5 |
| TH-45 |  | 0 |
| TH-46 |  | 18 |
| TH-47 | [MT446416](https://www.ncbi.nlm.nih.gov/nuccore/MT446416) | 22 |
| TH-48 |  | 0 |
| TH-49 | [MT446417](https://www.ncbi.nlm.nih.gov/nuccore/MT446417) | 5 |
| TH-50 | [MT446418](https://www.ncbi.nlm.nih.gov/nuccore/MT446418) | 9 |
| TH-51 |  | 16 |
| TH-52 |  | 5 |
| TH-53 | [MT446419](https://www.ncbi.nlm.nih.gov/nuccore/MT446419) | 14 |
| TH-54 | [MT446420](https://www.ncbi.nlm.nih.gov/nuccore/MT446420) | 11 |
| TH-55 | [MT446421](https://www.ncbi.nlm.nih.gov/nuccore/MT446421) | 14 |
| TH-56 |  | 0 |
| TH-57 | [MT446422](https://www.ncbi.nlm.nih.gov/nuccore/MT446422) | 8 |
| TH-58 | [MT446423](https://www.ncbi.nlm.nih.gov/nuccore/MT446423) | 1 |

Table S5. E. coli phages isolated in-house from sewage. GenBank accession numbers are indicated when appropriate. Supplemental file ‘plaque assay details.xlsx’ contains the detailed plaque plaquing data.

| *Phage name* | *HER ID* | *Phage Family* | *% bacteria producing plaques (out of 79)* |
| --- | --- | --- | --- |
| ΦX174 | 36 | *Microviridae* | 0 |
| PHI 1 | 255 | *Myoviridae* | 1 |
| Phi 2 | 256 | *Myoviridae* | 23 |
| Phi 3 | 257 | *Myoviridae* | 23 |
| Phi 4 | 258 | *Myoviridae* | 11 |
| Phi 5 | 259 | *Myoviridae* | 37 |
| Phi 6 | 260 | *Myoviridae* | 19 |
| Phi 7 | 261 | *Myoviridae* | 18 |
| Phi 8 | 262 | *Myoviridae* | 18 |
| Phi 9 | 263 | *Podoviridae* | 11 |
| Phi 10 | 264 | *Podoviridae* | 9 |
| Phi 11 | 265 | *Myoviridae* | 23 |
| Phi 12 | 266 | *Myoviridae* | 18 |
| Phi 13 | 267 | *Myoviridae* | 22 |
| Phi 14 | 268 | *Myoviridae* | 22 |
| Phi 15 | 269 | *Myoviridae* | 25 |
| Phi 16 | 270 | *Myoviridae* | 8 |
| 121Q | 128 | *Myoviridae* | 0 |
| BW-1 | 155 | *Myoviridae* | 11 |
| Esc-7-11 | 22 | *Podoviridae* | 1 |
| Haiti | 176 | *Myoviridae* | 0 |
| HER252 | 252 |  | 8 |
| HK243 | 139 | *Siphoviridae* | 3 |
| I2-2 | 213 | *Inoviridae* | 1 |
| K20 | 366 | *Myoviridae* | 14 |
| MED1 | 504 | *Microviridae* | 6 |
| N4 | 77 | *Podoviridae* | 4 |
| O103 | 337 | *Podoviridae* | 0 |
| P1kc | 93 | *Myoviridae* | 0 |
| PR64FS | 217 | *Inoviridae* | 0 |
| PR772 | 221 | *Tectiviridae* | 1 |
| RB69 | 158 | *Myoviridae* | 14 |
| SS4 | 364 | *Siphoviridae* | 0 |
| T2 | 25 | *Myoviridae* | 9 |
| T3 | 26 | *Podoviridae* | 5 |
| T4 | 27 | *Myoviridae* | 9 |
| T5 | 28 | *Siphoviridae* | 13 |
| T6 | 29 | *Myoviridae* | 13 |
| T7 | 30 | *Podoviridae* | 3 |

Table S6. E. coli phages obtained from the Félix d’Hérelle Reference Center for Bacterial Viruses at Université Laval. Supplemental file ‘plaque assay details.xlsx’ contains the detailed plaque plaquing data.


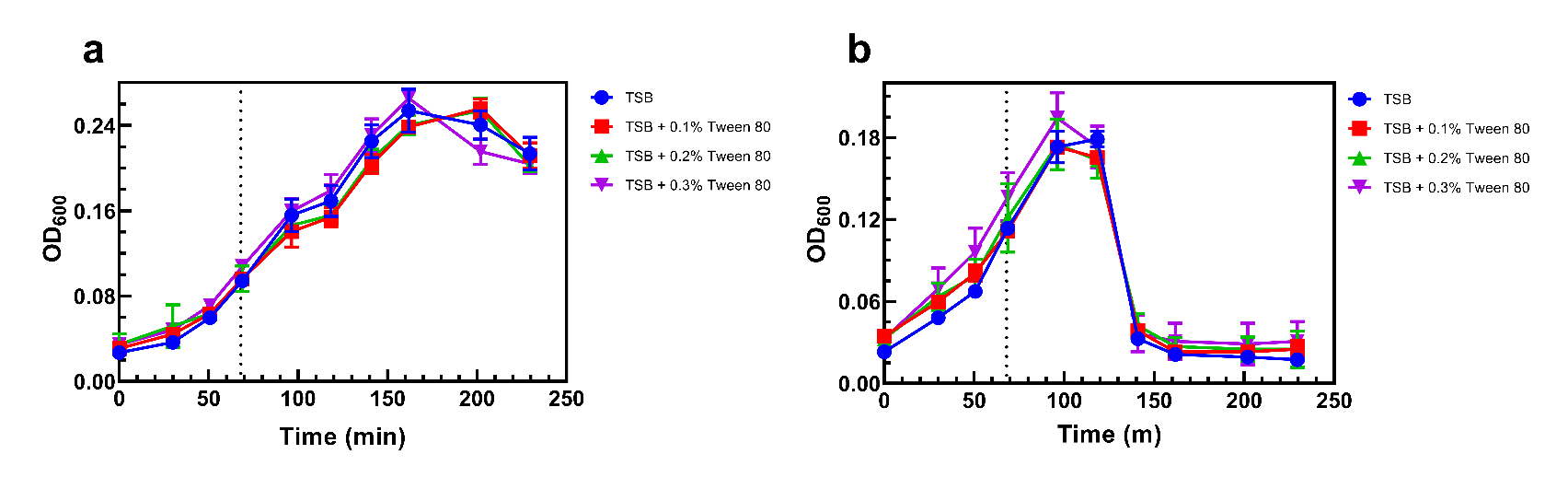


Figure S1. Effect of various Tween 80 concentration on (**a**) DH5α bacterial growth, and (**b**) T7/DH5α phage infection monitored through OD measurements at 600 nm.

Figure S2. Comparison of phage host range via plaque assay (prior to recombination with NanoLuc reporter) and via luminescence assay (after recombination with NanoLuc reporter) shows increased infection performance on 39 E. coli strains.


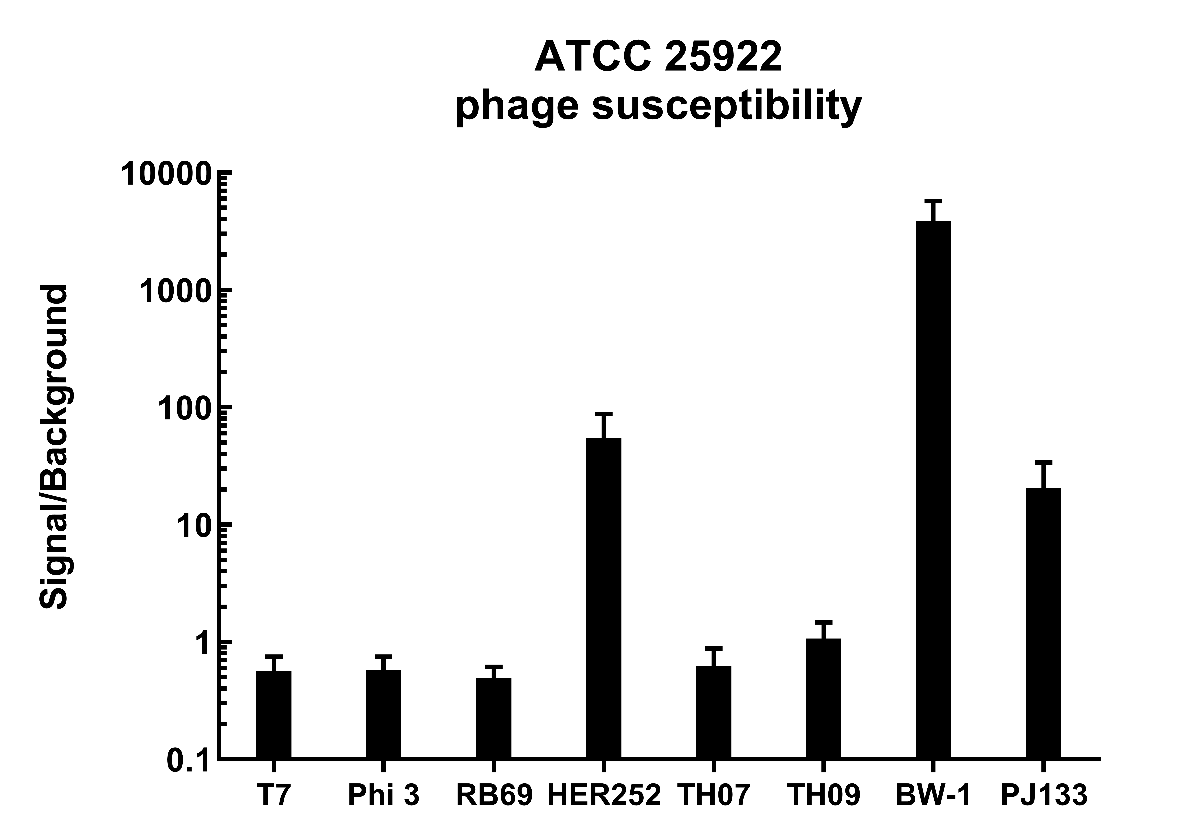


Figure S3. ATCC 25922 individual phage susceptibility.


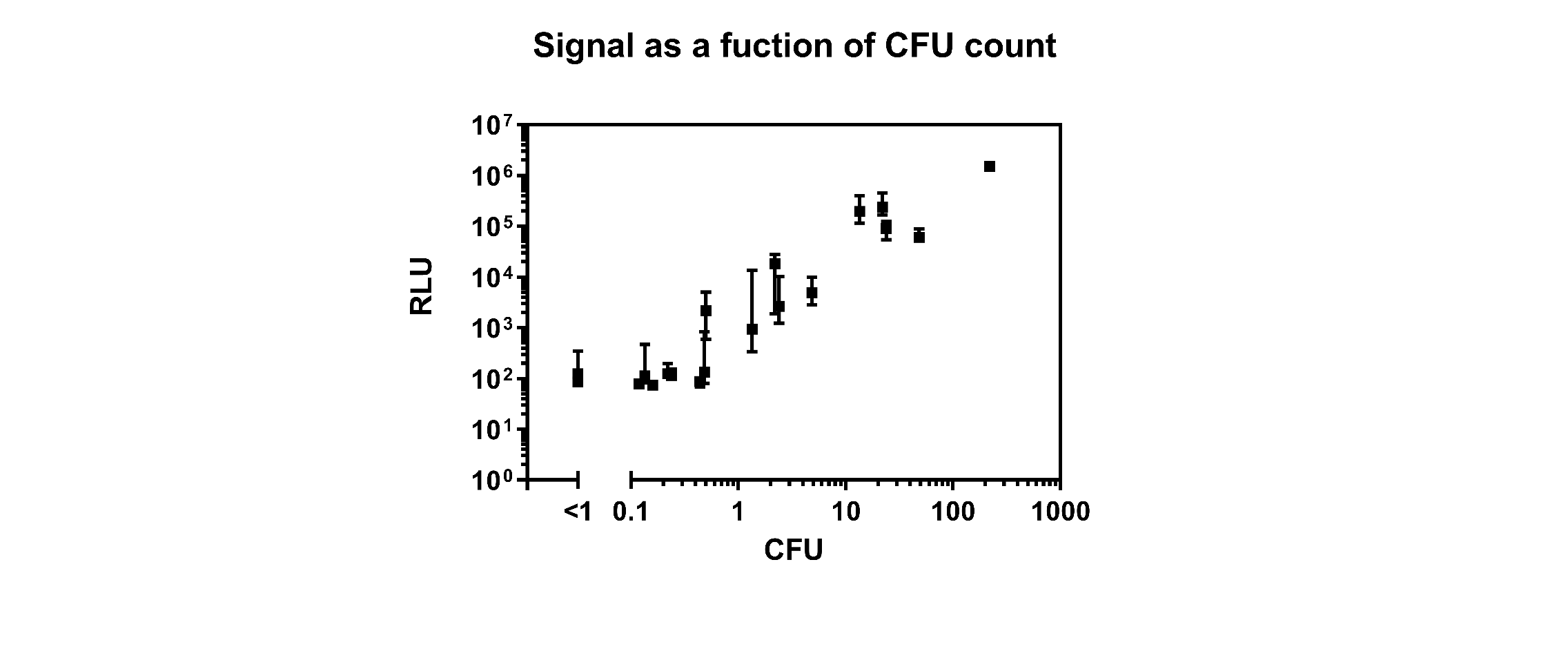


Figure S4. Luminescence signal as a function of CFU values as determined by membrane filtration field kit. Assay was performed in 96-well filter plate. Data points show median values of 16 to 48 replicates, and error bars show interquartile ranges.
